# Optimizing Immunomarking Systems and Development of a New Marking System Based on Wheat

**DOI:** 10.1673/031.011.8701

**Published:** 2011-07-11

**Authors:** Vincent P. Jones, Tawnee D. Melton, Callie C. Baker

**Affiliations:** Tree Fruit Research and Extension Center, Washington State University, 1100 N. Western Ave., Wenatchee, WA 98801

**Keywords:** *Cacopsylla pyricola*, *Choristoneura rosaceana*, *Lydia pomonella*, dispersal, ELISA, mark-capture

## Abstract

Immunomarking systems used to track large-scale movement patterns of insects are highly dependent on the efficiency of the enzyme linked imunosorbent assay (ELISA) reaction and logistical factors (e.g. concentration of marker applied, ability of the marker to wet the insect cuticle, and trapping methods). This paper examines ways to increase ELISA efficiency and mediate logistical factors, and provides information on a new immunomarking protein based on wheat gluten. The present studies on improving efficiency of the ELISA reactions showed that specially treated microplate surfaces were needed for soymilk and gluten assays, but not for egg albumin and casein assays. Sample dilution was investigated and was found to improve the signal/noise (S/N) ratio for the albumin and casein assays, but S/N ratios for the gluten and soymilk assays were less sensitive. However, for all assays, marked specimens were still detectable even with dilutions down to 6% of the original sample, which would allow more tests to be run on the same initial sample volume. For the logistical factors, these studies showed that marking of an insect by having it walk across a dried residue could be virtually eliminated for the casein and soymilk assays when the concentration applied was reduced to < 4%, but residues of 0.125% egg that had been aged in the field seven days still marked 37.5% of test insects placed on the residues. Also, the adjuvant Sylgard^®^ 309 used at 80 ppm enhanced wetting of the insect cuticle and had little or no effect on the ELISA reaction, but the wetting agents R-11 and Silwet^®^ L-77 were much more likely to negatively affect ELISA performance. Five different trapping adhesives were also evaluated and found to reduce ELISA efficiency 38–45% for the casein assay and 61–78% for the soymilk assay, while the albumin and gluten assays were unaffected. The information provided in this paper can be used to help correct for inherent differences in marking efficiency of the different proteins by manipulation of sample preparation, adjuvants, and concentrations applied.

## Introduction

Understanding landscape level movement patterns of insects promises to improve understanding of both population ecology and insect pest management. To date, most studies on insect movement have used mark-release-recapture techniques, using laboratory-reared insects that are marked using various methods and then released in the field (Hagler and Jackson 2001). Unfortunately, extrapolating movement patterns of laboratory-reared insects to naturally occurring ones is of dubious validity because of behavioral abnormalities that are common with inbred laboratory-reared colonies.

An immunomarking method useful for markrelease-recapture studies has been described previously (Hagler et al. 1992). That system used proteins not present in the agricultural systems (rabbit and chicken IgG) to mark insects by either applying the proteins to the insect exterior or by feeding the protein to laboratory-reared individuals. After recapture, the proteins were detected using enzyme linked immunosorbent assays (ELISA) specific to each protein. Unfortunately, the rabbit and chicken IgG proteins used were extraordinarily expensive (roughly $500/L), which precluded their use in marking large areas to study wild population movements.

However, from the standpoint of better understanding landscape level movement patterns, immunomarking has the potential advantage of allowing the use of multiple markers to measure movement between different areas. These studies also pointed the way in general to use proteins novel to a particular ecosystem for marking (Hagler et al. 1992).

The next major advance in immunomarking was the use of commercially available crude protein sources (egg white, soymilk, and casein) that could be diluted in water and applied using normal agricultural spray equipment or applied as dusts using dried versions of the proteins (Jones et al. 2006). These methods could be considered to be “second-generation” immunomarking protocols and are useful in both mark-release-recapture studies and mark-capture studies where the insects in the field are marked directly, eliminating concerns of behavioral anomalies related to use of laboratory-reared insects. To date, the second generation markers have been used with several insect species to quantify movement between areas marked with different proteins (Jones et al. 2006; Boina et al. 2009; Horton et al. 2009; Basoalto et al. 2010; Hagler and Jones 2010).

Using the second generation immunomarking systems in a mark-capture design is much more challenging than mark-release-recapture studies commonly used with first generation immunomarking (Jones et al. 2006). In part, this is a result of having to use water of highly variable quality to dilute the antigen (i.e. pH, water hardness, organic solids) and the difficulty of wetting the insect cuticle (which is hydrophobic) using typical spray equipment. However, even with those issues, it was found that insects can mark themselves within <5 minutes by walking on a fresh-dried residue (Hagler and Jones 2010) and that the mark can last up to 20 days (Boina et al. 2009). Studies have also shown that insects can mark themselves at levels >90% by walking across a 12 day—old residue of an egg marker on an apple leaf, but recovery of the mark from insects walking on casein or soymilk residues was considerably less efficient (Jones et al. 2006). Studies in citrus and cotton show that residual marking has similar trends, but that there are significant differences probably related to leaf surface texture, hairiness, and waxes present (Boina et al. 2009; Hagler and Jones 2010). Those studies all suggest that improving the residue or having an additive that could improve penetration into tight spaces and/or increase wetting of the cuticle would likely improve the usefulness of the immunomarking system in some situations. In addition, having a concentration of each marker that would result in marking by direct contact alone without residual marking would provide increased flexibility in experimental design and potentially reduce costs of some experimental protocols.

A concern specific to the immunomarking technique is that tested insects need to be captured separately to reduce the possibility of transferring the mark by contact. Previous studies used sticky traps to capture insects (Jones et al. 2006; Boina et al. 2009; Horton et al. 2009; Basoalto et al. 2010; Hagler and Jones 2010), but different types of sticky material may cause problems by competitive binding to the ELISA well by physically coating a portion of the insect and preventing the marker protein from being extracted, or by binding to the antibodies and causing false positives. The physical coating of the insect is particularly a problem with some of the polybutene sticky materials used in insect traps because they may wick up on the insect.

Finally, a major drawback of the immunomarking system is that the microplate wells used for ELISA assays can only bind from 220 to 620 ng/cm^2^ of protein (depending on the surface characteristics) (Esser 1997). Improper extraction techniques can result in a sample containing non-target proteins in much higher concentration than the desired marking proteins. When a sample solution with these characteristics is applied to an ELISA well, a low signal may result because concentrationdependent competition for binding sites between the target and non-target proteins (Crowther 2001). A low signal may also occur when using a clean sample (containing only the desired marking protein) present at very high concentrations if the secondary antibodies are unable to bind to the antigen because of steric inhibition (i.e. the marker molecules are too closely packed on the plate well for attachment of the antibodies) (Crowther 2001). Thus, dilution of a sample may result in better signal in some situations.

Optimizing the ELISA procedure, application methods, and trapping methods are critical for use of the immunomarking system. In this paper, the effects of: (1) microplate surface treatments on ELISA performance, (2) sample dilution on marker detection, (3) marker concentration on residual marking, (4) adhesives on ELISA reaction, and (5) agricultural spray adjuvants on the ELISA reactions were all examined. Finally, an assay for wheat flour was developed for use in immunomarking, which expands the currently available number of independent marks possible to four.

## Materials and Methods

### General ELISA protocols

The ELISA protocols were the same as those described by Jones et al. (2006) with the following modifications to the blocker solutions and antibody diluents to reduce cost. Phosphate buffered saline (PBS) (Sigma-Aldrich, www.sigmaaldrich.com) + 1300 ppm Silwet L-77 (Helena Chemical Co., www.helenachemical.com) + 20% bovine serum (BS) (Sigma-Aldrich) was used as the blocker for the egg and soymilk assays, and PBS + 20% BS was used for the casein assay. The primary antibody for the soymilk and casein assays was diluted in PBS + 1300 ppm Silwet L-77 + 20% BS, and for the egg assay was diluted in PBS + 30% BS.

In all studies, after incubation of the TMB (3, 3′, 5, 5′-tetramethylbenzidine) chromogen (ImmunoPure Ultra TMB substrate kit # 34028; Pierce Biotechnology, www.piercenet.com), 80 1 of 2 N H_2_SO_4_ was added to each ELISA well to stop the reaction. The stopped solution was then read using a dual wavelength microplate reader (Emax plate reader; Molecular Devices, www.moleculardevices.com) at 450 nm using 490 nm as the reference standard. All readings were corrected (blanked) using wells with the TBS (tris-buffered saline, pH 8.0, catalog number T-6664, Sigma Aldrich) + 0.3 g/L EDTA control (sodium (tetra) ethylenediamine tetra acetate, Sigma Aldrich) extraction buffer with no antigen. The use of dual wavelength to read the optical density (OD) and the correction using the sample object not exposed to antigen greatly reduces OD of the negative controls, but also reduces the likelihood of low-level non-specific binding resulting in a false positive. As measured by the microplate reader, the OD values range from 0 to 4, with the highest numbers indicating the darkest color and the highest concentration of antigen (marker protein).

### Wheat gluten ELISA protocols

The gluten assay was developed as an indirect ELISA and used essentially the same protocol as the soymilk assay as described above (all antibody diluents, blocker solution, wash protocols, incubation times and temperatures, same secondary antibody, sample extractions and volumes). The only exception was that a rabbit anti-gliadin primary antibody (Sigma Aldrich; catalog # G9144) was used instead of the soy primary antibody. The rabbit antigliadin primary antibody responds to gluten from wheat. The assay was tested to determine its sensitivity in the same manner as described by previously for the other three marker assays (Jones et al. 2006). Essentially, a serial dilution of a 10 ppm high-gluten wheat flour (Bob's Red Mill Natural Foods, www.bobsredmill.com) solution was tested to determine when all replicates (N = 8) of a given dilution were higher than the mean plus four standard deviations of the TBS + EDTA control. Sensitivity is presented as ppm of gluten, not gliadin.

### Effect of different microplate surface treatments

Microplates are manufactured with different surface treatments to enhance binding of antigens that have different chemical properties. Four different types of plates from the same manufacturer (Nalgene-Nunc International, www.nalgenunc.com) representing a range of surface treatments available were tested. These plates ranged from an untreated surface (cat. no. 260836), one that enhanced binding of hydrophobic antigens (Polysorp, catalog # 456529), one that has enhanced affinity for both hydrophobic and hydrophilic antigens (Maxisorp, catalog # 456537) to a surface that has an enhanced affinity for highly polar compounds (Multisorp, catalog #467340). All microplates tested had flat-bottomed wells.

Microplate tests were set up to evaluate binding of the different antigens (soymilk, casein, and albumin all at 1 ppm, gluten at 10 ppm) with the negative controls being pear psylla, *Cacopsylla pyricola* (Foerster) (Homoptera: Psyllidae), that were ground in 1 ml TBS + EDTA buffer using microtube grinders (USA Scientific Inc., www.usascientific.com). All plates for a given antigen were run the same day with identical positive and negative controls and used the same antibody solutions to reduce variation. All microplates were blanked on wells coated with only TBS + EDTA. Microplate performance for a particular antigen was evaluated by how well it bound the antigen as determined by the OD of the positive samples and how low the OD was for the negative control pear psylla samples. To summarize this information, the signal/noise ratio (S/N) was calculated as:


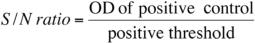


The denominator of the equation is actually the OD value required for a ground pear psylla to be considered marked using the mean OD + four standard deviations criterion (Jones et al. 2006). The ratio was not tested statistically for differences in the ratio, but instead used it as a broad guide as to the suitability of the different plate types for a particular marker antigen.

### Dilution effects on marker detection

Samples were collected into 1 ml of TBS + EDTA buffer and soaked for 3 minutes, the insect was then removed and the buffer tested for antigen. The insect was removed to prevent the extraction of large amounts of non-specific compounds which could cause non-specific binding or if the marker is in too high a concentration that would cause steric inhibition. Dilution was examined to reduce these potential problems, but a secondary benefit of dilution was the possibility that more tests could be run on the same volume of original sample if necessary.

The effect of diluting the sample on detectability and the S/N ratio was performed using 1:1 serial dilutions (using TBS + EDTA buffer) of positive samples that resulted in a range of concentrations from 100 to 1.56% of the original sample. The positive samples were obtained by spraying 2 ml of marker solution on arenas containing the test insects using an airbrush (Testors, www.testers.com). The rates of marker applied are given in tables 2 and 3. Test insects were adult obliquebanded leafroller, *Choristoneura rosaceana* (Harris) (Lepidoptera: Tortricidae), and *C. pyricola* adults that were either ground in 1 ml TBS +EDTA buffer using a microtube pestle or not. All samples were processed using the normal assay protocols.

### Effect of concentration applied on residual marking

As mentioned above, there are situations where it would be useful to have no residual marking. A logical method of reducing residual marking is to reduce the concentration of the marker applied. This approach is feasible because the concentrations applied in previous studies were >1 million times the detection limit of the different assays (Jones et al. 2006).

To mark insects in this study, water sprout shoots on full size apple trees (c.v. Red Delicious) at the Washington State University Tree Fruit Research and Extension Center (WSU-TFREC) in Wenatchee, WA, were dipped into 3.7 L Ziploc^®^ plastic bags filled with 1 L of marker solution. For each shoot, all leaves below the portion placed in the bag were removed and a piece of flagging tape was attached to the shoot that had the type of marker and concentration applied. All leaves on a shoot were collected one or five days after treatment and brought to the laboratory for testing. In the laboratory, a 7 mm leaf disc was removed with a cork borer from each leaf and submerged for 3 minutes in a microcentrifuge tube with 1 ml TBS + EDTA buffer solution. The leaf discs were then removed and the assays run on the buffer solution. The remaining portions of the leaves were used to line the inside a 1 L container along the sides, top, and bottom. Twenty-four pear psylla collected from unmarked pear trees at WSU-TFREC, were added to each container. After 24 hours, the insects were removed from the leaves, placed in 1 ml TBS + EDTA buffer for three minutes, discarded, and the buffer was tested for the marker. Because pear psylla rarely feed on apple leaves, and because the buffer tested had only washed the exterior of the insect (i.e. no grinding was involved), any psylla testing positive acquired the mark by contact with the treated surface. Three concentrations of each marker were initially tested (Table 1), but three additional concentrations of the egg marker were needed to reach the point that residual contact marking was minimized. In this second set of concentrations, the same methods were used, but leaves were collected 1 and 7 days after treatment. The gluten assay was not tested in this fashion because wheat flour does not readily dissolve in water; instead it becomes (at best) a suspension and does not dry uniformly, but leaves clumps of flour particularly where leaf hairs are most dense.

### Effect of different spray adjuvants in laboratory and field studies

The initial stage of this test was simply to determine if the spray adjuvants (Table 6) would speed the wetting of the cuticle for codling moth, *Cydia pomonella* L. (Lepidoptera: Tortricidae) adults. Each adjuvant was evaluated by placing a 2µ1 droplet of distilled water plus adjuvant at full manufacturer's field rate on a moth's wing. The wing was then observed under a dissecting microscope and classified as either: (1) showing wetting within 30 sec, (2) wetting within 5 min, or (3) not showing appreciable wetting. Only adjuvants that fell into category 1 were of interest and were tested further. For each adjuvant from category 1, a 1:1 serial dilution was performed down to the point that the adjuvant would no longer wet the cuticle of our codling moth adults within 30 sec.

The lowest concentration of the spray adjuvant that resulted in wetting of the wing was then tested for any inhibitory effects on the ELISA reactions. These inhibitory tests were run for the casein, albumin, and soymilk assays by mixing enough antigen with tap water to generate a 20000 ppm solution and then performing 1:1 serial dilutions with tap water to generate 10000, 5000, 2500, and 1250 ppm marker solutions. For the wheat flour antigen, because a 20,000 ppm suspension could not be made, the rates used were 15000, 7500, 3750, and 1875 ppm. Each of the serial dilution samples were split in half, and the adjuvant was added at the rates determined above to one of the two samples and the other sample served as a no-adjuvant control. The standard ELISA was then run to determine adjuvant effects on the ELISA reaction as determined by OD. The concentrations tested were chosen because they started at the highest concentrations used for field marking (except for the gluten assay) and decreased to levels that would simulate aged residues.

Data were analyzed using *t*-tests (unequal variance) to determine if the average OD of the antigen only positive control at a give ppm was significantly different than the adjuvant plus antigen at the same ppm. Because there were five concentrations tested for each antigen/adjuvant, the Bonferroni adjustment for the number of comparisons (i.e. α = 0.05/5) was used to insure type I errors were minimized (Quinn and Keough 2008).

### Effect of trapping adhesives

Trapping adhesives were tested by collecting a small amount (≈ 8 mg) from traps purchased from four different manufactures on toothpicks (Table 4). The toothpicks were then placed in a microcentrifuge tube for 3 minutes with 1 ml TBS buffer. After that point, the toothpicks were removed, and half of the buffer was used as a no antigen adhesive treatment and for the other half, 1 1 antigen was added to the solution to act as a positive antigen adhesive treatment. These samples were then run through a standard ELISA with separate negative (TBS buffer only) and positive treatments (TBS + antigen). A total of 8 replicates per treatment were run for each adhesive treatment. In addition to the trap adhesives, an adhesive (tangle trap liquid insect trap coating) designed to remain tacky, but essentially dry, was evaluated. This material was evaluated by brushing a small amount on the toothpick (∼ 8 mg), and after it set (1–2 min), it was run through the same assays. Analysis of the adhesive studies were done by using one-way ANOVA followed by Dunnett's test (Anonymous 2009) performed separately for positive and negative treatments with α = 0.01 for each assay.

## Results

### Gluten assay sensitivity

**Figure 1.**
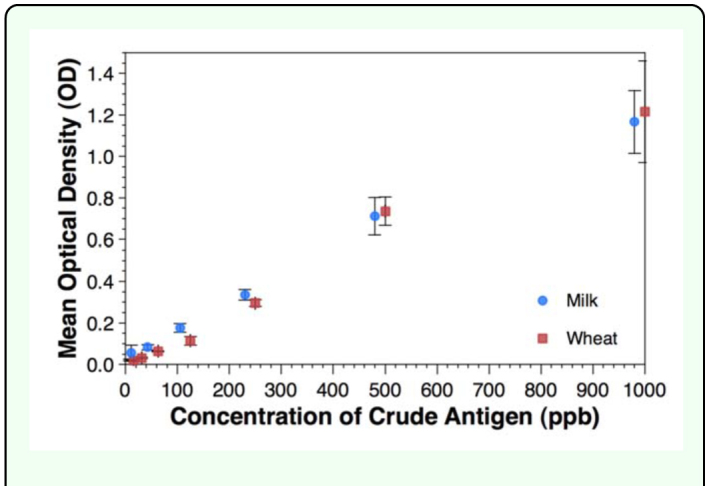
Effect of antigen concentration on optical density for the casein and gluten antigens. Error bars represent ± 1 SD. Values of the gluten concentration are offset 20 ppb to allow separation of data points. High quality figures are available online

The gluten marker was detected in all eight wells down to 15 ppb using the standard Polysorp microplates. The change in OD with antigen concentration was virtually identical to that seen for the casein assay (Figure 1).

### Microplate surface treatments

The four antigens each responded slightly differently to the microplate surface treatments. For the casein (cow's milk) antigen, the S/N ratio varied from 6.4 to 37.3. The Polysorp surface had the highest S/N ratio (37.3), followed closely by the Maxisorp surface (35.7) (Table 1). The other two types of microplates were markedly inferior in terms of the S/N ratio, primarily because of the low binding of the 1-ppm standards on those plates.

**Table 1.**
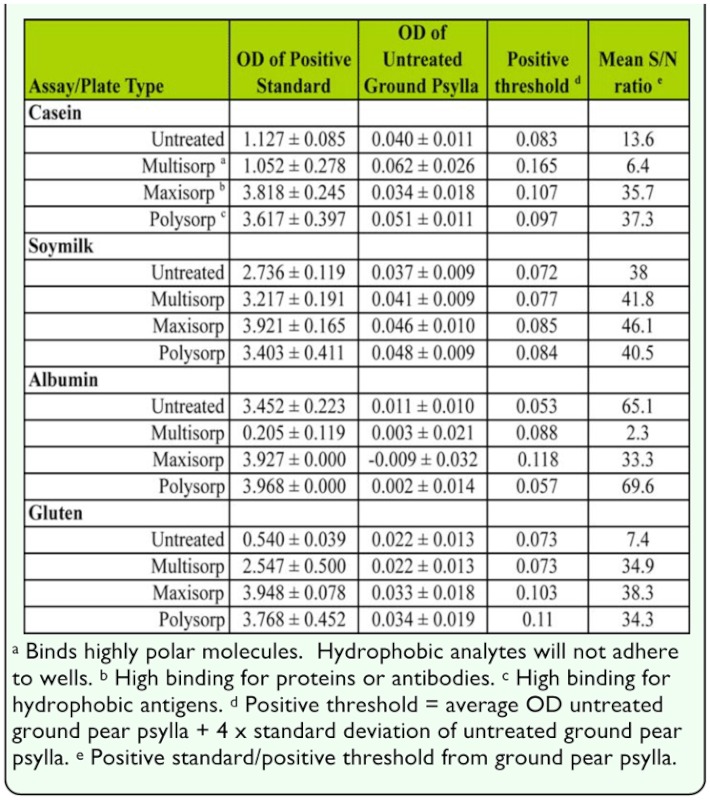
Effect of antigen concentration on optical density for the casein and gluten antigens. Error bars represent ± 1 SD. Values of the gluten concentration are offset 20 ppb to allow separation of data points.

The S/N ratios for the different microplate types with the soymilk antigen were relatively uniform, varying from 38 to 46.1. The best microplate type in terms of S/N ratio was the Maxisorp surface, with the Multisorp and Polysorp surface treatments being very similar, and the untreated surface treatment being only slightly lower. The better performance of the treated plates is primarily a result of a higher binding of the positive standards, which was greater than the increased OD found in the negative controls. The slight difference in performance between the different plate types would not alone justify the increased costs associated with the special surface treatments for the soymilk antigen.

Albumin showed large differences in S/N ratio between surface treatments, with the Polysorp and untreated plates being the top two and the Maxisorp and Multisorp having only 48 and 3.3%, respectively, of the S/N ratio obtained by the Polysorp plates. The low Multisorp plate performance is related to the poor binding of the 1 ppm standard, while the Maxisorp performance is related to its high variability in binding of the negative control ground pear psylla homogenate. As with the soymilk assay, the performance differences associated with the Polysorp versus the untreated surface plates would not justify the higher cost of using albumin.

The gluten antigen showed little difference between the Multisorp, Maxisorp, and Polysorp surface treatments in terms of the S/N ratio. However, the positive standard bound much better to the Maxisorp and Polysorp microplates than the other two types. Overall, there were few differences between any of the specialty surface treatment microplates for the gluten assay, but untreated microplates were unacceptable because of low binding of the positive samples.

### Dilution effects on marker detection

The serial dilution of the *C. rosaceana* samples marked with casein showed that all of the marked moths could be detected at all dilutions (Table 2). However, when the dilution dropped below 25%, the S/N ratio dropped from ∼ 70 to <40 with a rapid drop off as dilution of the sample increased (Table 2). The gluten assay had a very modest S/N ratio compared to the casein or albumin assays, but performed similar to the casein assay in that there was little difference in S/N ratio above 25% dilution, however, it dropped after that point. The soymilk assay showed the lowest S/N ratio of all the markers (Table 2). Dilution of the soymilk assay beyond 6.25% reduced the percentage of samples testing positive from 100 to 75%.

The albumin assay had by far the greatest S/N ratio of all the markers, in part because of the extremely low values of the positive threshold (Table 2). Dilution had the greatest impact on the S/N ratio of the albumin assay; the best performance for S/N ratio was between 25 and 6.25% dilution with the highest mean OD of treated moths registering with the 6.25% dilution. The increased S/N ratio and the higher positive control values between 25 and 6.25% dilution suggests that steric inhibition may be a factor with the albumin assay at high marker concentration.

**Table 2.**
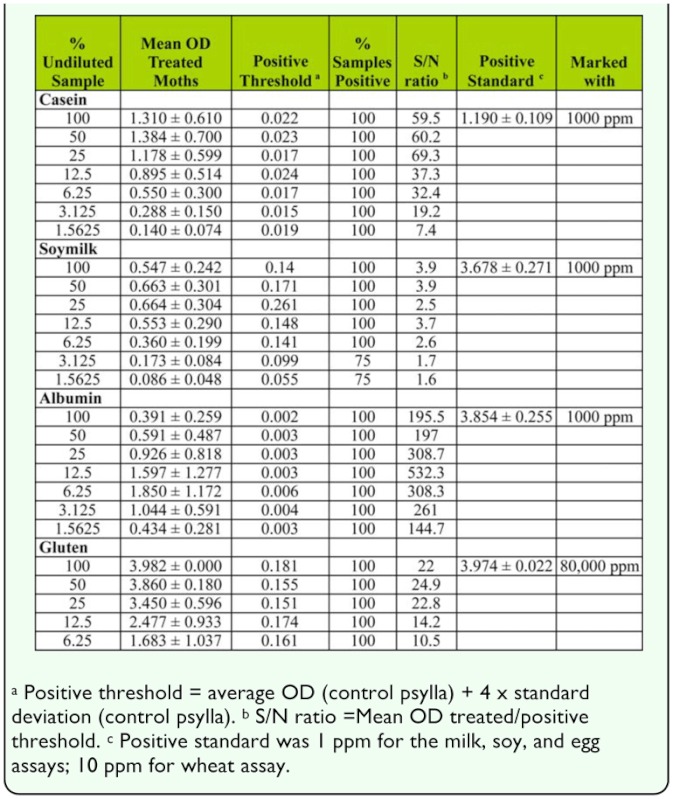
Effect of diluting samples of adult obliquebanded leafroller, *Choristoneura rosaceana* on the signal to noise ratio and the mean OD and the positive threshold (N=8).

The psylla samples illustrate the effect of having large amounts of non-marking proteins in a sample when grinding occurred (Table 3). In all cases, the negative controls were considerably higher and more variable in the ground samples than in the non-ground samples resulting in a much higher positive threshold and lower S/N ratio (Table 3). The extreme S/N ratio in the pear psylla samples controls (not ground) (Table 3) compared to the *C. rosaceana* (Table 2) is caused by the higher marker dose used with the experiments using pear psylla (20,000–80,000 ppm) compared to the dose used to mark the *C. rosaceana* (1,000 ppm for casein, soymilk, and albumin and 10,000 ppm with gluten).

**Table 3.**
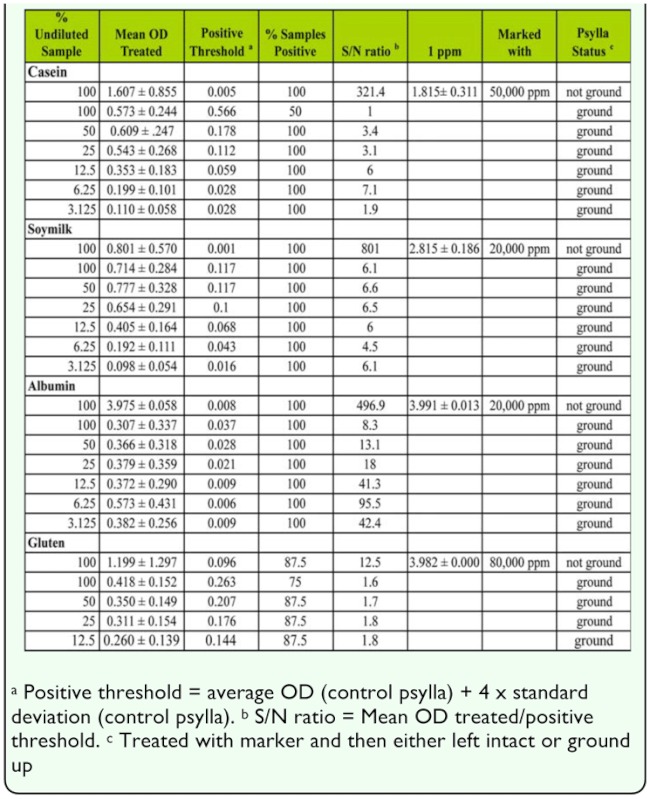
Effect of diluting samples with buffer on the signal to noise ratio and average OD of pear psylla *Cacopsylla pyricola* treated with marker and either ground or not (N=8).

For the casein assay, diluting the samples of pear psylla that had been ground up improved the ability to detect positive samples and optimal S/N ratio occurred at 12.5 and 6.25% of the original sample strength, however, the gains were relatively modest. The dilution of the soymilk samples had little effect at any of the dilutions tested; as the sample became more dilute, the positive threshold decreased, but so did the signal from the positive samples, making the S/N ratio fairly constant.

Similar to the results seen with *C. rosaceana,* pear psylla marked with casein showed that dilution increased the S/N ratio more than for any of the other marker proteins (Table 3). Part of this was the result of decreased positive thresholds, but at the same time the signal from the positive psylla controls increased as the dilution increased. Optimal concentrations were between 3.125 and 12.5% of the original sample strength.

**Table 4.**
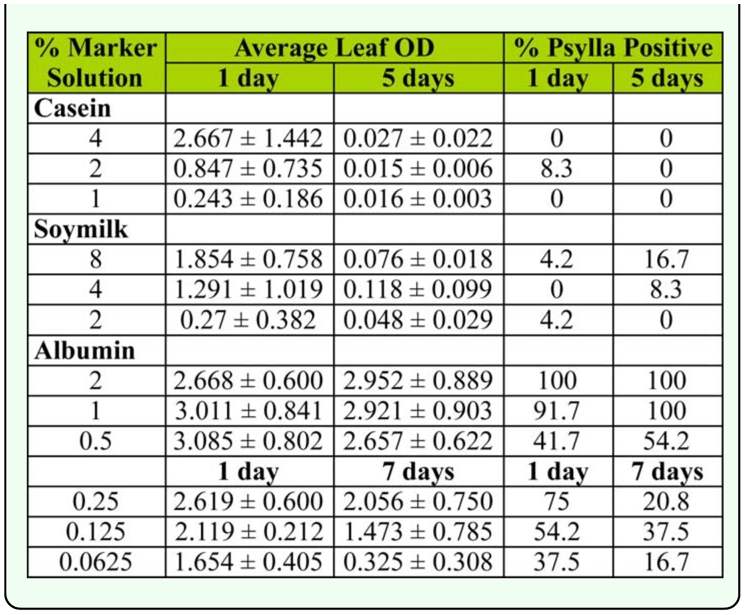
Effect of reducing the rate of antigen applied on the ability of pear psylla to walk across a dried residue and acquire a mark (N=24).

The gluten assay was the least sensitive to sample dilution with psylla samples marked with gluten. For dilutions between 50 to 12.5%, the S/N ratio remained between 1.6 and 1.8; the positive threshold decreased in the same ratio as the decrease in the signal from the positive psylla (Table 3). In contrast to the other markers, the gluten-marked psylla were never marked at 100%, regardless of the dilution. However, the percentage marking was similar across all dilutions. This suggests that while the sample could be diluted to allow more evaluations of a particular sample (e.g. for challenging the same sample with the different antibodies to detect multiple marks on a single specimen), it would not be an effective strategy to improve sensitivity of the assay.

### Effect of concentration on residual marking

Lowering the milk concentration applied to leaves resulted in lower leaf OD and reduced the ability of the psylla to acquire the mark by walking over a dried residue (Table 4). None of the psylla placed on the leaves that had been dipped in 1 or 4% milk solution acquired enough of the marker to read positive for either sampling day. Two insects (8.3%) placed on the leaves dipped in the 2% solution one day after treatment scored positive, but none placed on leaves collected five days after treatment tested positive.

Leaves dipped into soymilk solutions showed a pattern similar to the milk dipped leaves, where the marker was easily detectable on leaves dipped in the two highest concentrations on day one, but only two leaves read positive (in the 4% solution treatment) five days after treatment (Table 4). Psylla showed a very low percentage marking, particularly in the 2 and 4% treatments.

Psylla placed on leaves dipped in egg white solutions were marked at a much higher rate than those placed on either the milk or soymilk residues. In the initial set of concentrations, all leaves were highly positive, even 5 days after treatment (Table 4). In the 1 and 2% solution treatments, >90% of the insects were able to acquire the mark on both days. However, at the 0.5% rate, marking increase slightly from the one-day-old residue to the five-day-old residue. In the second set of concentrations the marking of the leaves was initially high and, even after aging seven days in the field, was high enough to mark psylla at the 0.0625% concentration at the same rate as the 8% solution of soymilk marker.

**Table 5.**
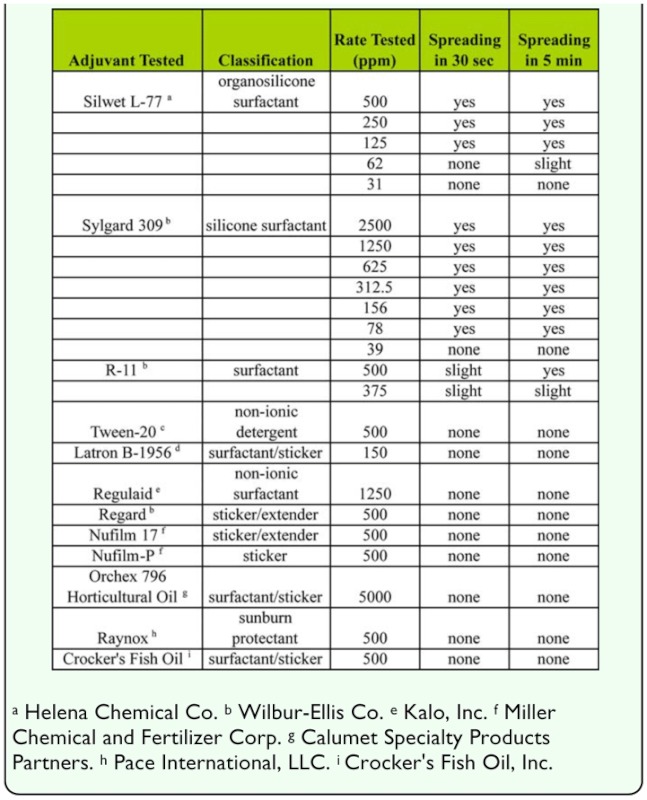
Evaluation of the ability of different spray adjuvants to wet codling moth *Cydia pomonella* adults when applied as 2 µl droplets to the wing.

### Spray adjuvant effects

The initial survey showed only three of the twelve spray adjuvants had the ability to wet the insect cuticle within five minutes (Table 5). The silicon surfactants Silwet L-77 and Sylgard 309 allowed the water to quickly spread within 30 s, and R-11 showed a slight spreading (Table 5). In the dilution series, Silwet^®^ L-77 would cause wetting within 30 s down to 125 ppm, but below that level wetting was greatly reduced. Sylgard 309 was effective down to 78 ppm, but not below that level. The R-11 surfactant showed no wetting below 375 ppm.

**Figure 2.**
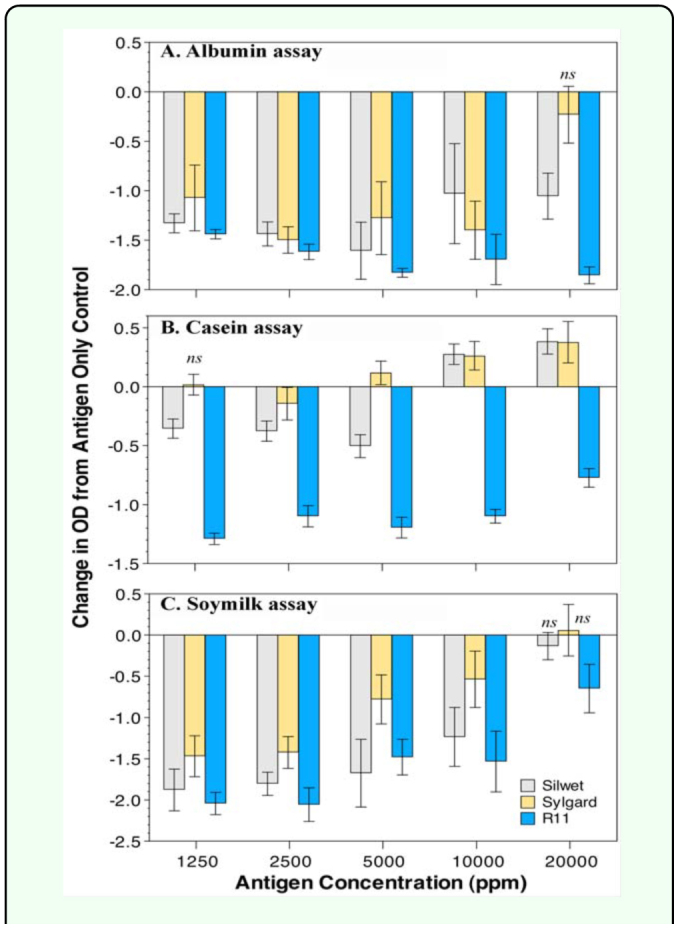
The effect of adding Silwet L-77, Sylgard 309, or R11 on OD at five different concentrations. (A) Albumin assay, (B) Casein assay, (C) Soymilk assay. Bars represent mean differences between antigen only (control) and antigen + adjuvant treatments; error bars represent 99% confidence intervals of the differences; all differences are significant at α = 0.05 (corrected for the number of comparisons) except those marked with *ns.* High quality figures are available online

Evaluation of the effect of the three spray adjuvants on the ELISA reaction showed that the addition of the R11 adjuvant always caused significant depression of OD compared to the control; the differences typically decreased as concentration of antigen increased, except in the egg assay (Figure 2, Table 3). Sylgard^®^ 309 at 80 ppm resulted in significant reductions in OD at low antigen concentrations for the albumin and soymilk assays, but had no significant effect on the casein assay at any antigen concentration. In fact, Sylgard^®^ significantly increased the OD at the top 3 casein antigen concentrations. The gluten wheat assay performed similar to the casein assay with only the top concentration showing a statistically significant depression in OD of ≈ 0.4 OD units. The albumin assay was most sensitive to Sylgard, with all antigen concentrations except for the highest showing significant reductions in OD. The Silwet L-77 treatment caused significant reductions in OD at the lower two antigen concentrations in all assays, but at the highest concentrations for the soymilk, casein, and gluten antigens there were no significant reductions in OD. In the albumin assay, Silwet L-77 always caused significant depression in the OD.

**Table 6.**
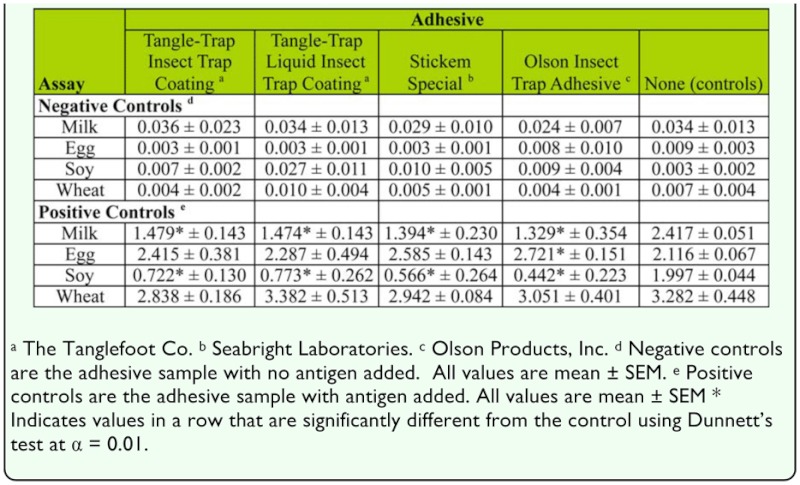
Effect of different trapping adhesives on ELISA reactions.

### Effect of trapping adhesives

The effect of adhesives used on traps varied depending on the assay. In the no antigen adhesive treatments, none of the adhesives resulted in significantly higher OD values for any of the assays (Table 6). In contrast, in the positive antigen adhesive treatments, the OD was significantly reduced for casein and soymilk compared to the no adhesive positive treatments (Table 6). In the casein assay, the reductions were similar among the adhesives and varied from 38 to 45% of the no adhesive positive treatments. The reductions were roughly twice as large with the soymilk assay, where they varied from 61 to 78% of the no adhesive positive controls. In the egg assay, the Olson Insect Trap Adhesive resulted in an OD significantly higher than the positive controls, but a lack of increase in the negative controls (no egg present) indicates that this not a result of the adhesive itself testing positive.

## Discussion

In many respects, the studies reported herein are similar to optimization studies of ELISA protocols in many different fields (Crowther 2001; Anonymous 2010). However, while guidance from ELISA optimization studies in other fields is valuable, that guidance is not always directly applicable to the specific assays used in immunomarking. In addition, use of these marking systems in the field has a different set of problems associated with them than laboratory-based clinical ELISA or mark-release-recapture studies (Jones et al. 2006; Horton et al. 2009; Hagler and Jones 2010).

The present study shows that the microplate surface treatments are an important component for immunomarking studies. While untreated microplates can be used for the soymilk or casein assays, surface treated microplates are crucial for sensitivity in the casein and gluten assays. This study only examined microplates from Nalgene-Nunc, but microplates from four other manufacturers were evaluated with similar results. An important concern is the variability between different batches of microplates (of the same type) from the manufacturer. When buying microplates, they can be either certified or uncertified, with the difference being that certified plates are sub-sampled for performance before packing and shipping (untreated plates are not available as certified). Our results suggest that the extra cost of certified plates (even if a special surface is thus used, as for soymilk or albumin) may be well worth the initial higher initial cost; multiple cases of plates (from different manufactures) over the past four years that had substandard performance and that needed to be returned support the worth of extra expenditure on certified plates. The relatively high labor cost to run the assays, as well as the loss of sample and time, outweighs the small difference in microplate cost.

The ability to dilute a sample and still obtain an accurate assessment of marking is a big factor when samples have large amounts of non-specific proteins that reduce the detectability of the marker through competitive inhibition. This problem is particularly severe when whole body homogenates are used; the same samples that are marked when just the surface of the insect is washed, are often classified as unmarked when whole body homogenates are used (Hagler and Jones 2010). Use of dilution may allow some of the same samples to be correctly classified because of greater sensitivity, but results will vary between the different antigen assays and the degree of concentration dependent non-specific binding occurring. In addition to helping mitigate the effects of competitive inhibition, dilution can also reduce the effects of steric inhibition where marker concentrations are high. The egg assay in particular benefitted greatly by dilution of the sample in both test insects. Finally, dilution is also valuable for extending the number of tests that can be run on a single sample, particularly if the experimental design requires the detection of multiple markers.

The dilution series also show the positive threshold (negative control OD + 4 × standard deviation of that control) is extremely low for the albumin assays and that dilution also helps reduce this value (Table 2 and 3). This may prompt concern that the abumin assay might be sensitive to higher rates of false positives. However, there are several mitigating factors that make this less likely than the numbers might indicate. First, there are two negative controls on each plate. The first set of controls is just the extraction buffer with no antigen present and this helps insure that the plate was not inadvertently contaminated; in conjunction with the positive control (antigen + extraction buffer) it signals that the assay was done correctly. The second set of negative controls is the extraction buffer + the sample type (e.g. untreated insects or leaves), that helps limit the importance of low-level non-specific binding associated with the sample organism/object. This second negative control is actually used for calculation of positive threshold to determine if the sample is marked or not. Thus, if the insect species tested has some sort of protein that causes a weak reaction, the positive threshold will reflect this contamination and reduce the likelihood of a false positive. Secondly, use of two wavelengths to measure the OD corrects for sample turbidity or scratches on the plate; this practice also reduces the OD reported and makes the positive threshold appear very low. Third, recent studies have shown ways to improve calculation of positive thresholds if multiple plates are used (Sivakoff et al. 2011), but these may or may not be applicable to a given situation. Perhaps the simplest way to resolve any concerns of falsely classifying a sample as positive is to use the lowest reliably detected dose for a given assay as a second positive control and to assume that all OD values less than that are unmarked, regardless of the positive threshold calculated by using the negative controls. The only downside of this is that the number of samples that can be run on a given plate is reduced by the addition of the second positive control.

The concentration of markers applied in the field for optimal marking depends heavily on several factors: (1) the adjuvants used in the spray, (2) the type of marking desired (contact only versus contact + residual), and (3) the type of trapping adhesives used. In terms of adjuvants, Sylgard 309 at 80 ppm could be used with higher rates of the markers for all of the assays. The data presented here is similar to previous work on apple and citrus where soymilk, albumin, and casein markers applied in the range of 10–20% with full rates of Sylgard 309 (apples) or Silwet L-77 (citrus) did not reduce residual marking (Jones et al. 2006; Boina et al. 2009). However, our results suggest that lower rates of the adjuvant would likely improve ELISA performance at least when the residues have weathered, or if lower rates of marker were used for other reasons.

The type of trapping adhesive is primarily an issue when the casein or soymilk assays are used, and can be at least partially corrected for by increasing the marker concentration. Differential marking can also be at least partially corrected for it in the analysis by using the percent of individuals marked with antigen X trapped inside the area treated with antigen X (e.g. the number of egg marked individuals in egg marked area) as an indicator of marking efficiency (Jones et al. 2006).

Our data also showed that by lowering the concentration applied, residual marking could be completely eliminated for the casein and soymilk marker systems. However, the egg marker is unsuitable for contact only marking because even at low rates significant marking occurred. The spray adjuvant studies showed that they would also reduce residual marking at low protein concentrations, perhaps enough that low rates of the egg marker combined with Silwet L-77 would eliminate or significantly reduce residual marking, while still marking individuals directly contacted by the spray.

The residual marking data clearly show that the casein and soymilk assays require a higher initial marker concentration to perform similarly to the albumin assay. In the present studies, twenty percent soymilk or milk were typically used as the marker solutions versus 10% for the egg marker; it may be beneficial in certain circumstances to increase the milk and soymilk concentrations to compensate for adhesive effects and to increase the residue if needed. The likely reason for of the difference in residual marking between the albumin assay and the soymilk and casein assays is that the antibodies for soymilk and casein are actually reacting to only a small portion of the crude antigen, whereas the antibody to egg albumin, which makes up virtually all of the material applied (liquid egg whites). For example, milk is composed of ≈ 3.2% protein and casein is ≈ 75% of that total. This means that a 20% milk solution is actually only ≈0.5% casein. Unfortunately, pure casein cannot be used in the same manner because it is insoluble in water, and is also more costly.

The gluten assay provides another marker system that is useful in evaluating movement patterns of insects. Wheat is probably best used when a dry marking system is desired because it does not go into solution well; instead it acts more as a suspension requiring care when adding it to a liquid spray system. If possible (bearing in mind phytotoxicity concerns), Sylgard 309 should probably be used if wheat is applied as a liquid to help wet the flour particles. The benefit of the wheat system is that its cost per kg is relatively low compared to soy flour or powdered milk, and especially low relative to powdered eggs.

Overall, immunomarking is still one of the few ways to mark wild insects in the normal environment on any meaningful scale. However, there are multiple factors associated with the ELISA reaction that need to be considered when designing a mark-capture study in the field. This study provides information that helps optimize the marking procedure reported previously (Jones et al. 2006). However, our results clearly suggest that the doses used in a particular crop/target insect system need to be established by specific studies because of the differences in surface texture of both the crop and target insect and behavior of the target insect. Depending on the studies being performed, higher concentrations or greater application frequency may allow the user to obtain useful information, but if large areas are being treated, specific residual marking studies should be performed to reduce the costs of experiments.
